# Neuronal Nitric Oxide Synthase and Post-Translational Modifications in the Development of Central Nervous System Diseases: Implications and Regulation

**DOI:** 10.3390/molecules28186691

**Published:** 2023-09-19

**Authors:** Cristina Maccallini, Rosa Amoroso

**Affiliations:** Department of Pharmacy, University “G. d’Annunzio” of Chieti-Pescara, Via dei Vestini 31, 66100 Chieti, Italy; rosa.amoroso@unich.it

**Keywords:** neurodegenerative disorders, nitric oxide, nNOS, nNOS inhibitors, post-translational modifications, PSD95-nNOS interaction inhibitors

## Abstract

In the Central Nervous System (CNS), Nitric Oxide (NO) is mainly biosynthesized by neuronal Nitric Oxide Synthase (nNOS). The dysregulated activation of nNOS in neurons is critical in the development of different conditions affecting the CNS. The excessive production of NO by nNOS is responsible for a number of proteins’ post-translational modifications (PTMs), which can lead to aberrant biochemical pathways, impairing CNS functions. In this review, we briefly revise the main implications of dysregulated nNOS in the progression of the most prevalent CNS neurodegenerative disorders, i.e., Alzheimer’s disease (AD) and Parkinson’s disease, as well as in the development of neuronal disorders. Moreover, a specific focus on compounds able to modulate nNOS activity as promising therapeutics to tackle different neuronal diseases is presented.

## 1. Introduction

**Figure 1:**
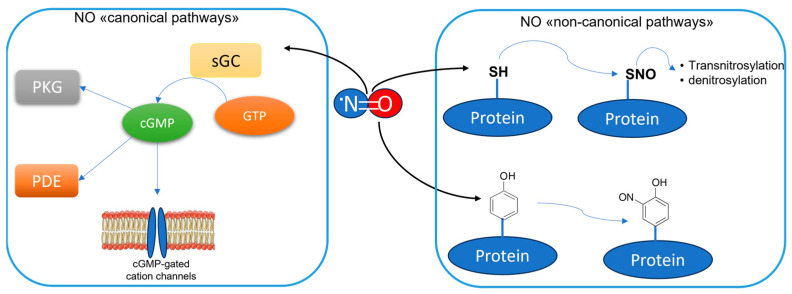
NO signaling pathways in the brain. The “canonical pathways” of NO signaling are initiated through the stimulation of soluble guanylate cyclase (sGC). The produced cGMP mediates downstream signaling mainly by interacting with PKG, PDE, and cGMP-gated cation channels. The “non-canonical pathways” of NO signaling involve the proteins’ post-translational modifications. Mainly, they are protein nitrosylation, which can lead to the transnitrosylation of other proteins as well as to their denitrosylation by denitrosilases, and protein nitrotyrosination.

The enzyme neuronal Nitric Oxide Synthase (nNOS) is an oxidoreductase primarily expressed in the nervous system and it is responsible for the conversion of L-Arg into L-Citr and Nitric Oxide (NO). This is a pleiotropic molecule, playing essential roles in mammalians [1]. In the nervous system, it mediates neuronal signaling as an unconventional neurotransmitter, since it is a gaseous molecule, not stored in synaptic vesicles but synthesized on demand by neurons [2,3]. Moreover, NO is a radical molecule, which can react with a variety of biological substrates both by nitrosylation, e.g., the direct reaction of NO with a reactant, or nitrosation, e.g., the indirect transfer of NO to biological nucleophiles via nitrosonium ions (NO^+^), which are preliminary generated from NO oxidation [4]. Under oxidative stress conditions, NO can react with the superoxide anion (O^2−^), thus forming Reactive Nitrogen Species (RNS), such as the peroxynitrite (ONOO^−^), which can generate potent oxidant intermediates responsible, for example, for proteins’ nitration. In any case, NO can regulate the neuronal physiologic and pathologic responses by finely tuning multiple pathways. Basically, they can be categorized as “canonical pathways” and “non-canonical pathways” (Figure 1). The first group are initiated by the nitrosylation of the metalloenzyme soluble guanylate cyclase (sGC), which catalyzes the production of cyclic guanosine monophosphate (cGMP) from triphosphate guanosine (GTP) [5,6]. Then, cGMP mediates downstream effects mainly through the interaction with protein kinase G (PKG), phosphodiesterase (PDE), and cGMP-gated cation channels [7,8]. NO can affect its downstream targets also through “non-canonical pathways”, i.e., inducing post-translational modifications (PTMs) of various proteins (Figure 1). In particular, S-nitrosylation, e.g., the reaction of NO with reactive cysteine thiols to form nitrosothiol groups, is a determining mechanism of NO-mediated signaling in the brain [9]. Being the primary source of NO in the Central Nervous System (CNS), nNOS is implicated in both physiological and pathological responses of neuronal cells [10,11]. Besides nNOS, there are also the endothelial NOS (eNOS) and the inducible NOS (iNOS), which are two other homolog NOS isoforms, playing a different role in the brain. In fact, eNOS is almost exclusively expressed in the vascular endothelial cells where it regulates vascular homeostasis [12], while iNOS is poorly found in some specific regions of the healthy brain, such as the hypothalamus, thalamus, and the amygdaloid nuclei, but is largely expressed mainly in the microglia after pro-inflammatory stimuli, as part of the innate immunity response [13,14]. The overexpression of iNOS is a major feature in different CNS diseases associated with neuroinflammation, such as Alzheimer’s disease (AD), Parkinson’s disease (PD), and Huntington’s disease (HD) [15], as well as in several psychiatric illnesses [16]. The consequent overproduction of NO is responsible not only for neurotoxicity due its conversion in the above-mentioned RNS, but it is also implicated in the loss of blood–brain barrier (BBB) integrity, inducing its increased permeability to different substances [17]. It is interesting to note that specific drugs for the therapy of AD, such as donezepil [18], or long-term treatment with the antidepressants fluoxetine or vortioxetine [19] can prevent the overexpression of iNOS connected to the oxidative stress observed in animal models of AD. The implications of the excessive production of NO by iNOS in the development of neuroinflammation and its specific role in CNS diseases have been extensively studied in recent years and they have been reported elsewhere [20,21].

In this review, given the prominent role of nNOS-derived NO in the brain, we briefly revise the main implications of dysregulated nNOS in the initiation and progression of the most prevalent CNS neurodegenerative disorders, i.e., AD and PD, as well as in the development of neuronal disorders. Moreover, a specific focus on compounds able to modulate nNOS activity as promising therapeutics to tackle different neuronal diseases is presented.

## 2. The Overstimulation of nNOS in CNS Disease Development

**Figure 2:**
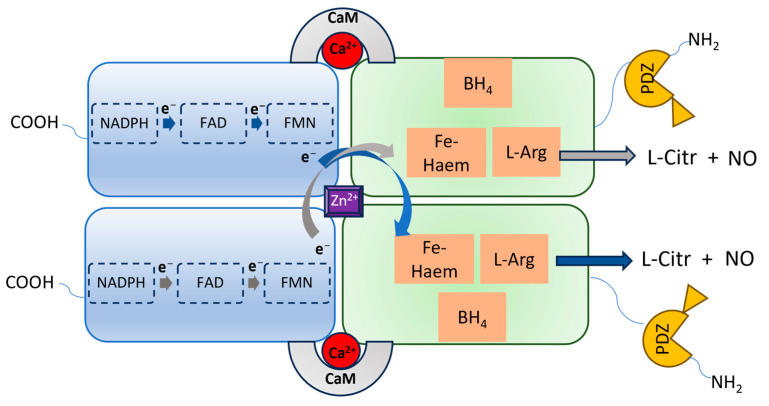
Representation of the nNOS homodimeric architecture. Each monomer has a carboxy-terminal reductase domain and an amino-terminal oxygenase domain, containing the L-Arg binding site and linked to the PDZ domain. In the reductase domain, electrons via the NADPH-FAD-FMN transport chain reach the heme iron of the opposite monomer oxygenase domain, facilitated by CaM, enabling the oxidation of L-arginine to L-citrulline, accompanied by the release of NO.

nNOS shares a similar architecture to eNOS and iNOS, being a homodimer in which each monomer contains an *N*-terminal oxygenase domain hosting the catalytic site, and a reductase domain containing the cofactors binding sites (Figure 2). The two domains are connected by a Ca^2+^-calmodulin (CaM) binding sequence and, specifically for nNOS, the oxygenase domain is also connected to the postsynaptic density protein, discs-large, ZO-1 (PDZ) domain, which is characterized by a β-hairpin sequence. Through the PDZ domain, nNOS can interact with other proteins, triggering different cascades of protein–protein interactions [22]. This also determines the specific subcellular localization and functions of nNOS. Indeed, nNOS can be found both in the neurons’ cytosol as well as bound to the postsynaptic membrane through the interaction with the postsynaptic density protein-95 (PSD-95)-NMDAR complex, or with other different proteins, such as Capon, syntrophin, or postsynaptic density-93 (PSD-93) [23,24,25]. These proteins regulate the activation of nNOS and its physiological functions.

In physiological conditions, the stimulated N-methyl-D-aspartate receptors (NMDARs) mediate the intracellular influx of Ca^2+^, which can bind the CaM sequence of nNOS, initiating its activity. The produced NO can behave as an anterograde or retrograde neurotransmitter, regulating memory, learning, and synaptic plasticity [26,27,28]. Indeed, nNOS-derived NO induces the full expression of c-Fos, Egr-1, Arc, and brain-derived neurotrophic factor (BDNF), which are key proteins associated with neuroplasticity. This was observed both in cortical cultures after bicuculline-evoked synaptic activity, and in in vivo mice models of experience-dependent plasticity in the whisker barrel cortex [29,30]. In particular, the signaling pathways involved in this effect include GMP, PKG, extracellular-signal-regulated kinase (ERK), and calcium–calmodulin (CaM)-dependent protein kinase II (CaMKII) [29].

**Figure 3:**
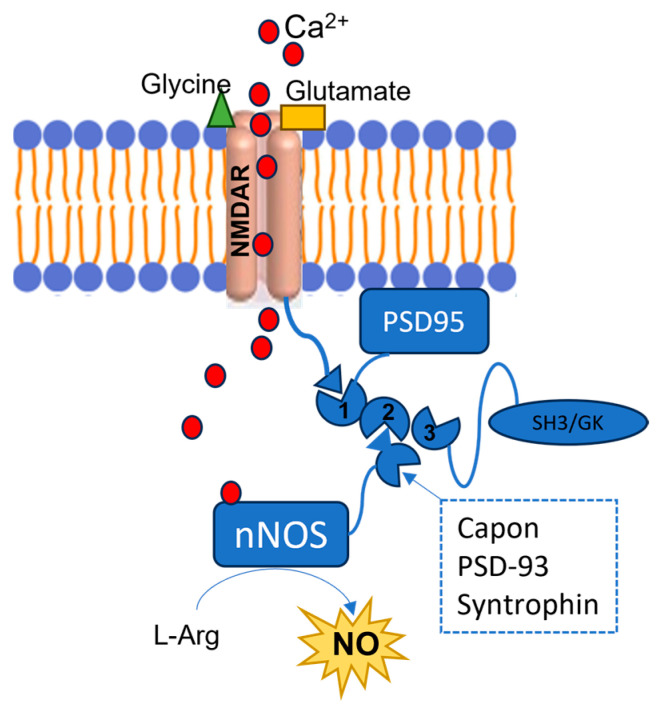
Representation of the NMDARs-PSD-95-nNOS complex. The red circles represent the Ca^2+^ ions, the green triangle represents a glycine molecule, and the yellow rectangle represents a glutamate molecule. NMDARs are bound to the PDZ1 domain of the PSD95, and when they are stimulated by the interaction with glycine and glutamate, they allow Ca^2+^ intracellular influx. This cation binds the nNOS CaM, and the enzyme interacts with the PSD95’s PDZ2 domain by its PDZ β-hairpin motif or with other proteins such as Capon, synthropin, or PSD-93. As a consequence, the enzyme’s activity is stimulated, although an excessive nNOS activation is observed in different SNC diseases, due to the NMDARs’ overstimulation.

However, in the early phases of pathological conditions such as stroke, AD, and PD, as well as in other neuropsychiatric conditions such as epilepsy and autism, an overactivation of NMDARs can be observed, followed by an excessive Ca^2+^ influx, with a loss of its homeostasis (Figure 3). These events lead to the prolonged overstimulation of nNOS, due to excessive intracellular Ca^2+^ levels [31,32]. The overproduced NO contributes to the development of such diseases by mediating proteins’ PTM, such as nitrotyrosination (Tyr-NO_2_), i.e., the reaction of tyrosine residues with ONOO^−^, and cysteine nitrosylation (SNO) (Figure 1). In the specific diseases’ context, these PTMs are mostly pathological, leading to proteins’ loss- or gain-of-function.

### 2.1. NO-Mediated PTM and Alzheimer Disease

**Figure 4:**
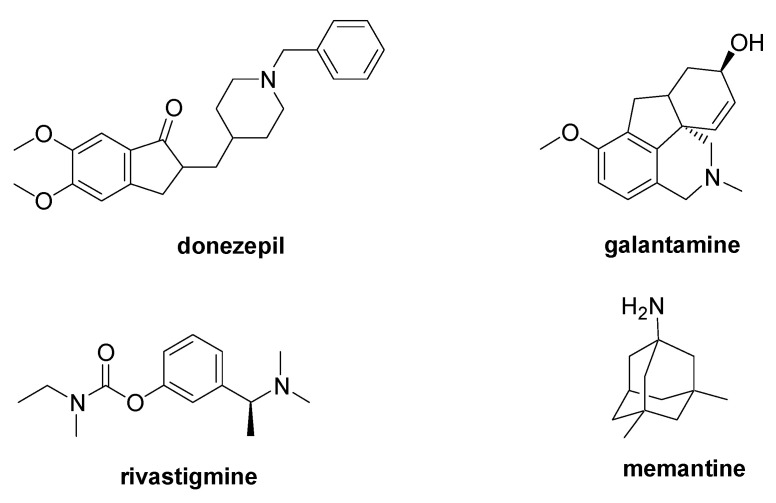
Chemical structure of the FDA-approved drugs for AD therapy.

AD is a progressive type of dementia characterized by the aggregation into β-sheets of the amyloid β peptide (Aβ), which is derived from the cleavage of the amyloid precursor protein (APP). Further biological markers of this disease are the intracellular neurofibrillary tangles caused by the excessive phosphorylation of the tau protein. The only FDA-approved AD medications are the acetylcholinesterase inhibitors (AChEIs) donepezil, galantamine, and rivastigmine, and the NMDA antagonist memantine (Figure 4) [33]. Despite the many clinical trials, no new drug has received FDA approval in the last 20 years, and, among the reasons of the clinical trial failures, the inadequate comprehension of the pathophysiology of the AD is a widely accepted explanation [34,35].

In general, cerebral regions of AD patients, specifically the hippocampus and the cerebral cortex, display higher Tyr-NO_2_ levels, and there is a positive correlation between nNOS expression and neurofibrillary tangles in neurons, as well as amyloid plaque accumulations and nitrergic neurons [36,37,38]. Moreover, the activity of gamma-secretase is affected by nitrotyrosination [39]. Together with β-site APP cleaving enzyme type 1 (BACE1), gamma-secretase is responsible for the production of Aβ_1–40_ or Aβ_1–42_ peptides. In AD patients, it has been demonstrated that the nitrotyrosination of this enzyme leads to the imbalanced production of Aβ_1–42_, which is more prone to aggregate and toxic species [40]. Furthermore, Aβ_1–42_ nitrotyrosination was also demonstrated to increase aggregates’ stability and toxicity [41]. In AD, the nitrotyrosination of important neuronal metabolic enzymes is also observed, such as lactate dehydrogenase and triosephosphate isomerase (TPI), with a reduction in their activity and important metabolic changes [42]. It was reported that nitro-TPI contributes to intracellular neurofibrillary tangle formation [42].

Besides nitrotyrosination, the extensive S-nitrosylation of proteins is also crucial for synaptic function and neuronal survival and it is associated with AD development [43,44]. Indeed, SNO protein modifications may induce further protein misfolding and neuronal and synaptic damage, leading to mitochondrial stress. In safe cells, these modifications are reversible thanks to the presence of antioxidants such as glutathione and de- and trans-nitrosylating enzymes [45,46]. However, this balance is compromised in the developing AD due to the excessive NO released from the overstimulated nNOS, which leads to massive SNO-proteins. For example, the S-nitrosylation at Cys 83/157 of Cyclic-dependent kinase 5 (CDK5), which is responsible for the cleavage of p35 to p25, upregulates the kinase activity, leading to dendritic spine loss and neuronal apoptosis [47,48]. It was observed that S-nitrosylation of the insulin-degrading enzyme (IDE) inhibits its activity to degrade Aβ [49], while S-nitrosylation of vesicular acetylcholine transporter (VAChT) and vesicular glutamate transporter 1 (VGLUT1) worsens the ACh turnover [50].

In addition, aberrant transnitrosylation reactions, i.e., transfer of the NO group from one protein to another, are highly implicated in AD synaptic loss due to activation of the alternative biochemical network to the physiologic functions of the involved enzymes [51].

### 2.2. NO-Mediated PTM and Parkinson Disease

**Figure 5:**
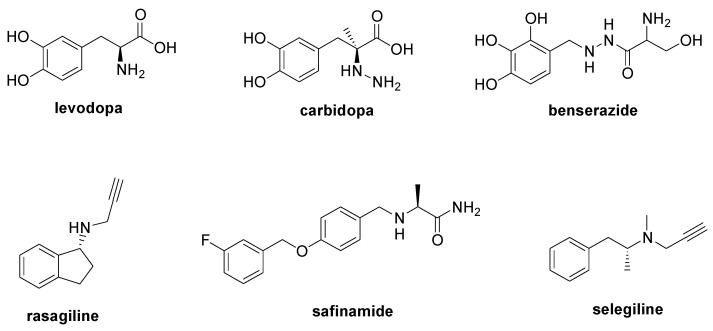
Chemical structures of approved drugs used in the management of PD.

PD is a degenerative condition of the brain associated with motor symptoms (rigidity, tremor, bradykinesia, and postural instability) and non-motor disorders (apathy, depression, cognitive dysfunction, and sleep disorders). The main cause of PD is the loss of dopaminergic neurons in the substantia nigra [52] due to the aggregation of the α-synuclein, a protein that regulates the trafficking and release of neurotransmitter vesicles [53]. Currently, there is no therapy to modify the course of PD, with its treatment only being palliative to alleviate the motor and non-motor symptoms that occur during the disease’s development. The administration of the levodopa (Figure 5) is considered the principal therapeutic approach to restore dopamine levels [54], and it can be combined with carbidopa or benserazide (Figure 5), two decarboxylase inhibitors useful to increase levodopa bioavailability [55]. Moreover, the simultaneous treatment with monoamine oxidase B inhibitors such as rasagiline, safinamide, and selegiline is recommended to increase dopamine levels (Figure 5) [55]. Entacapone and tolcapone, two catechol-O-methyltransferase inhibitors, are also used to promote the gastrointestinal absorption of levodopa [55].

Although the loss of dopaminergic neurons is a well-established mechanism involved in PD, the reasons leading to the initiation of this process are still unknown. Different studies have reported the accumulation of 3-nitrotyrosinated proteins and a neuronal upregulation of nNOS in cells isolated from PD patients [56,57,58].

It was demonstrated that α-synuclein nitrotyrosination induces its aggregation and inhibits its interaction with dopamine vesicles [59]. Moreover, the nitration of tyrosine hydrolase (TH) seems to be implicated in PD onset. This enzyme is involved in catecholamine synthesis from tyrosines, and its activity is impaired through nitration, lessening dopamine availability [60]. On the contrary, it was reported that TH nitrosylation at Cys 279 enhances its enzymatic activity both in vitro and in vivo, confirming the important role of NO in the subtle regulation of proteins involved in PD progression [61]. It was reported that the overproduced NO is responsible for the S-nitrosylation of different, other proteins, such as the disulfide isomerase and microtubule-associated protein 1b, as well as of CDK5, impairing axo-dendritic function and neurite length [62]. Moreover, the S-nitrosylation of parkin, a protein involved in the degradation of specific substrates, reduces its activity, and consequently neurotoxic proteins can accumulate, leading to ER stress [62]. These proteins’ PTMs alter network connectivity, which is associated with cognitive decline and neuronal death.

### 2.3. NO-Mediated PTM and Neurological Disorders

NO plays an important role in neurodevelopment, and its altered signaling appears implicated in the progression of a variety of neurodevelopmental and neuropsychiatric diseases. NO can either facilitate or suppress synaptic plasticity, depending on the brain area, concentration, and cellular environment [11,63]; therefore, both overactivation and downregulation of nNOS can be implied in the development of such diseases.

Recently, interesting studies have put in light connections between the specific genetic mutation of the Shank3 gene occurring in autism, a condition associated with deficits in communication and social skills, and excessive NO synthesis, which is responsible for aberrant protein nitrosation and S-nitrosylation [64,65,66,67]. Elevated levels of different SNO proteins functionally involved in the synaptic vesicle cycle, neurotransmission, and glutamatergic pathway, such as protein phosphatase catalytic subunit α-Ppp3ca, syntaxin-1a, vesicle-associated membrane protein 3, and others, were found in Shank3 KO mouse models [66,67]. Collectively, these observations provide insights into the specific pathological role of dysregulated NO production in autism spectrum disorders.

Epilepsy is the most common neurological disease and it was reported that nNOS-derived NO is neurotoxic in the epileptic brain, due to the formation of peroxynitrite after its reaction with the superoxide radical, triggering PTZ kindling epilepsy-induced neural damage [68]. Indirect evidence suggests that the inhibition of nNOS in pilocarpine-induced temporal lobe epilepsy mice can protect against hippocampal neuronal injuries by increasing neuropeptide Y expression, which has been implicated in energy homeostasis and neuroprotection [69]. Moreover, overexpression of NO and lipid peroxidation was reported in the brain of pentylenetetrazol (PTZ)-induced epilepsy rats, and antioxidant treatment normalized their levels [70]. Specific NO-mediated PTMs are implicated in epilepsy; for example, NO is responsible for type 1 ryanodine receptor (RyR1) S-nitrosylation, inducing Ca^2+^ release from the endoplasmic reticulum through the Ca2 + release channel, and worsening the disease progression [71].

In an animal model of epilepsy, the kainate receptor, specifically glutamate ionotropic receptor kainate type subunit 2 (GluK2), undergoes S-nitrosylation, and SNO-GluK2 further potentiates calcium influx [72].

## 3. Inhibitors of nNOS as Modulators of CNS Disease Development

A potential therapeutic approach to rebalance the dysregulated NO signaling occurring in neuronal diseases is the inhibition of nNOS activity [73]. This can be achieved using two different pharmacologic tools: nNOS inhibitors and inhibitors of the PSD95-nNOS interaction.

### 3.1. nNOS Inhibitors

**Figure 6:**
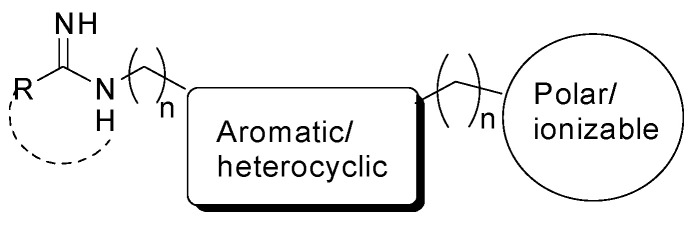
General pharmacophoric structure of an nNOS inhibitor.

In the last two decades, several compounds able to inhibit nNOS activity have been reported [74]. Since they compete with the natural substrate L-Arg for the catalytic binding site, the very first generation of NOS inhibitors were aminoacidic analogs of L-Arg, but they were unselective, blocking all the three NOS isoforms with potential severe side-effects. Therefore, non-aminoacidic substrate analogs were developed containing some structural requirements (Figure 6). In particular, they show a molecular moiety able to mimic the guanidino group of L-Arg, which is anchored to the highly conserved Glu residue in the enzyme catalytic site by means of bidentate H-bondings [75]. This moiety is linked to a rigid central core, which is usually aromatic and/or heterocyclic, and can give interactions with the heme protoporphyrin [76]. Then, there is a final tail, which usually contains a polar and/or ionizable group able to extend out of the catalytic site, where there are the major aminoacidic differences between the three NOS isoforms [77]. Among the other derivatives, sterically hindered amidines [78], thienylcarbamidines [79], indazoles [80,81,82], aminopyridines [83], and aminoquinolines [84] gave promising results as nNOS inhibitors, although further efforts are required to modulate these compounds’ pharmacokinetic properties. Indeed, a limiting factor in the clinical evaluation of nNOS inhibitors is their high polarity and basicity, which prevent their blood–brain barrier permeability.

**Figure 7:**
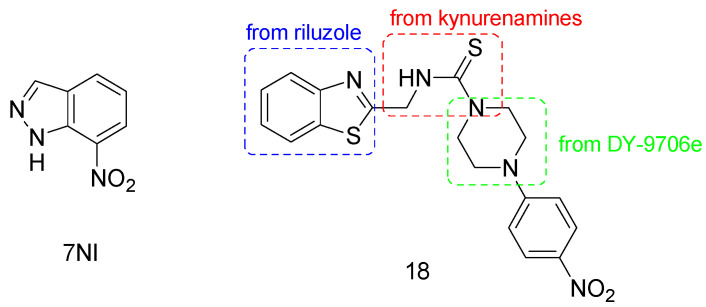
Chemical structure of nNOS inhibitors.

Thanks to its tissue specificity and good pharmacokinetics, 7-Nitroindazole (7-NI, Figure 7) has been studied in different models of neuronal diseases. It has been reported that short-term 7-NI treatment can prevent the acute degeneration of dopaminergic neurons caused by different neurotoxins [85,86]. Moreover, this compound is able to slow down the progressive neurodegeneration in a Parkinson’s disease in vivo model [87]. Very recently, 7-NI was also used to demonstrate the pathological role of NO in autism development, and the in vivo administration of this compound to a Shank3 mouse (M1) model of autism disease led to the recovery of a normal behavioral phenotype [67].

Recent promising results in the management of PD neurodegeneration were obtained by the new nNOS inhibitor 18 (Figure 7) [88]. It is a benzothiazole derivative containing a piperazine-1-carbothioamide moiety, designed by means of a hybridization approach of compounds directed against molecular targets involved in the progression of PD, i.e., pramipexole and riluzole, kynurenamine-based nNOS inhibitors, and the calmodulin inhibitor DY-9706e [89,90]. It was found that 18 allows the recovery of motor and non-motor functions in an induced unilaterally lesioned rat model of PD. Moreover, this compound modulated the oxidative stress markers, leading to increased levels of dopamine and lessening glutamate and nitrite ion levels.

### 3.2. PSD95-nNOS Interaction Inhibitors

The subcellular localization and activity of nNOS are influenced by the interaction of its PDZ domain with different proteins bearing this moiety as well (Figure 3) [10]. In particular, the PSD95-nNOS interaction is crucial for synaptic plasticity [24] and it is considered a potential biological target to modulate the overactivation of nNOS occurring in neuronal diseases [91]. In fact, the PSD95 can also bind the NMDARs by means of its PDZ1 domain, and if they are overstimulated, as in many neurodegenerative diseases, they allow the influx of high amounts of Ca^2+^ into neuronal cells, triggering the nNOS interaction with PSD95’s PDZ2 and its consequent overactivation. In particular, the nNOS-PDZ domain interacts with the PSD95-PDZ2 domain by means of its β-hairpin motif (Figure 2 and Figure 3) [11,92]. Therefore, peptides and small molecules have been designed to prevent the PSD95-nNOS interaction, either by binding the PDZ2 domain of PSD95 or by targeting the nNOS-PDZ domain.

**Figure 8:**
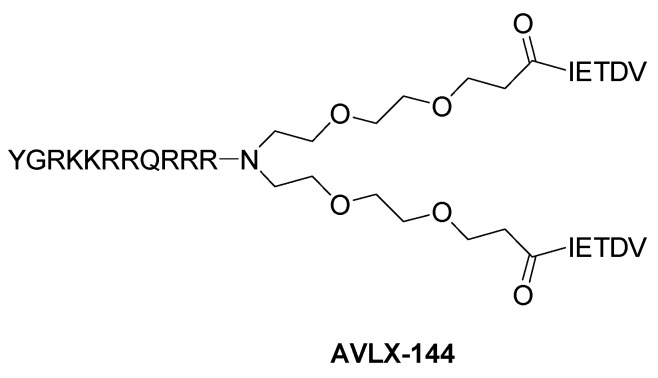
Structure of the peptide-based PSD95-nNOS interaction inhibitor AVLX-144.

AVLX-144 (Figure 8) is a peptide-based molecule able to bind the PSD95’s PDZ1 and PDZ2 domains simultaneously, blocking the protein binding to both NMDAR and nNOS, with a Ki of 4.6 nM. AVLX-144 is able to permeate the BBB and has proven to be neuroprotective in mice with focal ischemic brain damage [93]. Moreover, it has also been used in a model of cortical spreading depression, a condition associated with stroke and migraine aura, inducing an improvement in neuronal depolarization [94]. Further, AVLX-144 significantly reduced neurodegeneration, cytoskeletal degradation, peroxynitrite formation, and astrogliosis in an in vivo model of epilepsy [95]. Currently, this compound is under clinical development by Avilex Pharma (Copenhagen, Denmark).

To limit the potential drawbacks due to the potential interactions of these inhibitors with the PDZ domain of other proteins, new cyclic peptides based on the nNOS β-hairpin motif were designed, aiming to optimize the binding with the PSD-95 through more specific interactions [96]. Through the identification of the critical residues in the cyclic nNOS β-hairpin peptide responsible for maintaining high affinity to the PSD-95-PDZ2, two nonproteinogenic AA cyclic peptides were developed with an approximately 100-fold increased binding affinity compared with the wild-type peptide.

**Figure 9:**
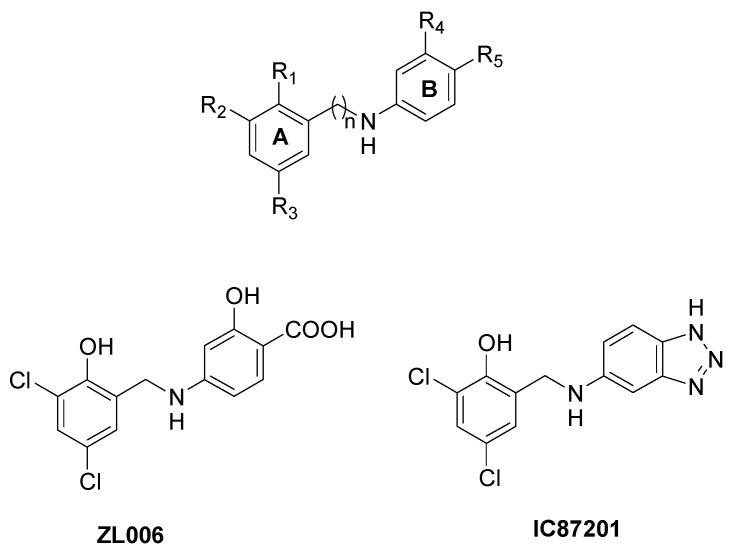
Chemical structure of small molecules able to selectively bind the nNOS β-finger and of ZL006 and IC87201.

Small molecules directed toward the nNOS β-hairpin motif have also been reported, in order to specifically avoid its interaction with PSD95. Starting from the observation that the nNOS β-finger adopts a suitable conformation to bind the PDZ2 domain of PSD95 only if an internal salt bridge between Asp62 on the nNOS PDZ domain and Arg121 on the nNOS β-finger domain is formed, a series of compounds showing hydrophobic ring A and hydrophilic ring B bearing a carboxyl group were prepared (Figure 9) [97]. Ring A forms hydrophobic interactions with Leu107 or Phe111 on the β-finger, hindering the conformational change of nNOS PDZ, while the carboxyl group on ring B interacts with Arg121, disrupting the intra-nNOS salt bridge. In particular, compound ZL006 (Figure 9) was the most potent nNOS-PSD95 inhibitor (IC_50_ = 0.082 µM), not affecting NMDA receptor function, nNOS expression, or nNOS catalytic activity [97]. ZL006 inhibited NMDA-receptor-mediated NO synthesis and was neuroprotective in neurons and animal ischemic stroke models [97]. Moreover, ZL006 attenuates hemorrhage-induced thalamic pain in mice, it reduces Aβ_1–42_-induced neuronal damage and oxidative stress, and it lessens neuronal injury and apoptotic cell death in MPP+-cultured cortical neurons [98,99,100].

By means of a high-throughput screening approach, another nNOS-PSD95 inhibitor was discovered (IC87201, Figure 9), although its interaction mode has not been fully elucidated [101,102]. This compound has shown great effects in animal models of ischemic stroke, depression, pain, and regenerative repair after stroke [101,103,104].

## 4. Conclusions

The dysregulated activation of nNOS in neurons is critical in the development of different conditions affecting the SNC. As a downstream event of NMDAR overactivation, an excessive production of NO by nNOS is responsible for a number of proteins’ PTMs, which can lead to aberrant biochemical pathways, impairing SNC functions. In this review, we revise evidence of how nitrotyrosination and cysteine nitrosylation, which are the two major NO-dependent PTMs, are related to the development of different SNC pathological conditions. Moreover, the promising effects that the pharmacological regulation of nNOS activity could have in the management of such diseases are also discussed. However, despite this evidence and due to the multifaceted role of NO both in SNC physiology and pathology, it remains crucial and challenging to understand the chronology of NO-induced PTM in the disease progression. Indeed, the nNOS’ physiologic activity should be preserved due to its role in neuroplasticity, long-term potentiation, and memory, while its overactivation should be tuned by means of molecules endowed with protein and tissue selectivity. In this regard, nNOS-mediated PPIs are supposed to be safer with respect to nNOS inhibitors, since the latter have been associated with side-effects due to their poor isoform selectivity and induction of iNOS [105,106]. In fact, blocking nNOS-PSD95 interactions should avoid any unwanted inhibition of eNOS.

However, more knowledge of the pathophysiology of proteins’ PTMs is needed to hopefully find new effective and safe treatments able to restore an appropriate NO signaling in SNC diseases.

## Data Availability

Not applicable.
